# Supplementary material for: Mapping the evolution of fertility support policies in China: A content and instrumental analysis
Source: PLoS One. 2025 Oct 9;20(10):e0332137. doi: 10.1371/journal.pone.0332137 (PMC12510515; doi:10.1371/journal.pone.0332137)
Supplement: S1 Appendix — (ZIP) [file pone.0332137.s001.zip › S1 Appendix. 226 original policy documents/49-国家医疗保障局办公室关于做好支持三孩政策生育保险工作的通知(FBM-CLI-4-5054450).doc]

国家医疗保障局办公室关于做好支持三孩政策生育保险工作的通知

**发布部门：** [国家医疗保障局](https://www.pkulaw.com/law/chl?Aggs.IssueDepartment=60431) [机构沿革](javascript:void(0))

**发文字号：**医保办发〔2021〕36号

**发布日期：**2021.07.06

**实施日期：**2021.07.06

**时效性：** [现行有效](https://www.pkulaw.com/law/chl?Aggs.TimelinessDic=01)

**效力级别：** [部门工作文件](https://www.pkulaw.com/law/chl?Aggs.EffectivenessDic=XE0303)

**法规类别：** [机关工作综合规定](https://www.pkulaw.com/law/chl?Aggs.Category=00301)

国家医疗保障局办公室关于做好支持三孩政策生育保险工作的通知
（医保办发〔2021〕36号）

各省、自治区、直辖市及新疆生产建设兵团医疗保障局：
　　为贯彻落实党中央关于优化生育政策促进人口长期均衡发展的任务部署，积极支持三孩生育政策落地实施，确保参保女职工生育三孩的费用纳入生育保险待遇支付范围，各地医保部门要按规定及时、足额给付生育医疗费用和生育津贴待遇，切实保障参保人员生育保障权益。同步做好城乡居民生育医疗费用待遇保障和新生儿参保工作。
　　各地医保部门要高度重视，提高政治站位，积极采取管用高效措施办法，确保三孩生育待遇政策落实到位，主动做好正向宣传，增强参保群众获得感。遇有重大情况和问题，及时向国家医保局报告。

国家医疗保障局办公室
　　2021年7月6日

© 北大法宝：（[www.pkulaw.com](https://www.pkulaw.com/)) 专业提供法律信息、法学知识和法律软件领域各类解决方案。北大法宝为您提供丰富的参考资料，正式引用法规条文时请与标准文本核对。
欢迎查看所有[产品和服务](https://www.pkulaw.com/helps/2.html)。法宝快讯：[如何快速找到您需要的检索结果？](https://www.pkulaw.com/helps/70.html)[法宝](https://www.pkulaw.com/helps/69.html)V6有何新特色？

扫描二维码阅读原文

**原文链接：**<https://www.pkulaw.com/chl/be55477596a64918bdfb.html>
